# Prevalence and patterns of multimorbidity among adults in rural Shanxi Province, China: A post-hoc exploratory subgroup analysis of a cross-sectional study

**DOI:** 10.1371/journal.pone.0330935

**Published:** 2025-09-10

**Authors:** Shuai Tang, Yanxing Li, Runlan Li, Hongmei Yang, Tianyou Hao, Maoyi Tian, Xinyi Zhang, Xiangxian Feng, Zhifang Li

**Affiliations:** 1 School of Public Health, Shanxi Medical University, Taiyuan, China; 2 Academy of Medical Sciences, Shanxi Medical University, Taiyuan, China; 3 School of Nursing, Changzhi Medical College, Changzhi, China; 4 School of Public Health, Changzhi Medical College, Changzhi, China; 5 Department of Prevention and Health Care, Affiliated Heping hospital of Changzhi medical college, Changzhi, China; 6 School of Public Health, Harbin Medical University, Harbin, China; College of Medicine and Health Sciences, United Arab Emirates University, UNITED ARAB EMIRATES

## Abstract

**Background:**

In China, the prevalence of chronic diseases is increasing, especially in rural areas, affecting younger populations and associating with multimorbidity. However, in resources-limited rural areas, there is a lack of primary data to the prevalence and patterns of multimorbidity in young populations. This study aims to analysis the differences in multimorbidity prevalence and patterns across different age groups and genders among adults in rural Shanxi Province.

**Methods:**

This study was a post-hoc analysis of a completed cross-sectional study. All the participants included in this post-hoc analysis were randomly selected from 80 villages from Shanxi Province. Multimorbidity, defined as the coexistence of two or more diseases in same individual, was assessed by collecting primary data (questionnaires and physical examinations) and routine data (health insurance claims and hospital electronic records).

**Results:**

A total of 2,208 participants were included, with a mean age of 57.7 ± 10.5 years. 1283 (58.1%) were aged 30–59 years and 1152 (52.2%) were females. There were 1524 cases (69.0%) with disease and 818 cases (37.5%) with multimorbidity. Hypertension had the highest prevalence (43.5%) as well as multimorbidity pattern was often associated with it (75.3%). The three most common dyad multimorbidity patterns were hypertension and stroke, heart disease, and chronic digestive diseases, respectively. Among 30–59 years, it was hypertension respectively and stroke, chronic digestive diseases, and arthritis, while among ≥60 years, it was hypertension respectively and stroke, heart disease, and diabetes. In males, it was hypertension respectively and stroke, heart disease, and chronic digestive diseases, while in females it was hypertension respectively and arthritis, chronic digestive diseases, and diabetes.

**Conclusion:**

The multimorbidity prevalence among adults in rural Shanxi Province is notably high. The pattern of multimorbidity is distinct differences between age groups and genders, suggesting that prevention and management of priority diseases in specific populations should be targeted.

## Introduction

With changes in lifestyle, an aging population, and increasing life expectancy, the prevalence of chronic diseases has been steadily rising [1]. Due to the prolonged course and complex etiology of chronic diseases, the coexistence of multiple chronic conditions has become the “norm” [2,3]. The World Health Organization (WHO) defines the simultaneous presence of two or more chronic health conditions in the same individual as “multimorbidity” [4]. Studies show that the global prevalence of multimorbidity is approximately 37.2% [5], while in the adult population in China, the prevalence is 36.3% [6]. Furthermore, the overall multimorbidity prevalence is expected to continue rising in the future, with this trend becoming more pronounced over time [7]. Multimorbidity may lead to multiple adverse health outcomes, including disability, cognitive impairment, and death [8,9], and may also increase the difficulty of treating and managing the disease, as well as the consumption of healthcare resources [10].This not only affects the quality of life of the patients but also places substantial burden on socio-economic development, the distribution of healthcare resources, and the functioning of public health systems [11–13].

In China, the majority of multimorbidity studies rely on secondary data analysis from large cohort studies, such as China Health and Retirement Longitudinal Study(CHARLS)and China Kadoorie Biobank(CKB) [14]. These studies primarily use self-reported data, which may be influenced by reporting bias, potentially leading to an underestimation of multimorbidity prevalence [15]. Secondly, most multimorbidity research has focused on urban populations, neglecting the circumstances in resource-limited rural areas [16]. This is particularly concerning because rural areas are disproportionately affected by multimorbidity due to a severe shortage of healthcare resources and limited health awareness among the population [17,18]. Finally, Although multimorbidity is more prevalent among the older population [19], the rising trend in its prevalence among middle-aged individuals is more pronounced [20,21]. But current studies in China largely concentrates on older multimorbidity patients, overlooking the younger population [6].

It is precisely because existing research suffers from a lack of rural primary and young population data as well as insufficiently accurate data. Therefore, this study utilizes data from a cross-sectional survey conducted among rural populations aged 30 and above in China, with the disease information complement with the routinely collected data, to reduce the affect of the report bias in order to robustly understand the prevalence and patterns of multimorbidity in rural areas of Shanxi Province.

## Methods

This study is a post-hoc subgroup analysis utilizing partial data (Shanxi Province) from a cross-sectional study registered with the China Clinical Trial Registration Center (ChiCTR2300069860) [22]. Ethical approval was obtained from the Harbin Medical University Ethics Committee (No. HMUIRB2022005PRE). Prior to the enrolment, all participants completed written informed consent.

### Participants and sampling

A total of 80 villages were selected from two counties (Lucheng District and Huguan County) in Shanxi Province. Residents aged 30 years above from the selected villages were invited to participate in this study. The recruitment process involved randomly selecting participants from a stratified list of villagers by gender and age groups (30–59 years, ≥ 60 years).Participants who are pregnant, unable to provide informed consent or unwilling to participate, unable to communicate effectively to complete the questionnaire, and unable to have blood and urine collected will be excluded.

### Definition of multimorbidity

This study follows the World Health Organization’s (WHO) definition of multimorbidity, which refers to the co-existing of two or more chronic diseases or health conditions [4]. The disease information in the study is primarily based on self-reported data from study participants (confirmed by secondary or higher-level hospitals), supplemented by routine data from local healthcare institutions’ electronic health records and the infectious disease surveillance system of the local disease control and prevention center, to compensate for any information that may have been omitted due to reporting bias in face-to-face questionnaire surveys. All routine data are standardized using the International Classification of Diseases, Tenth Revision (ICD-10). Using participants’ unique ID numbers, we linked and integrated data from these different sources. Finally, all disease conditions extracted from routine data were uniformly mapped to the 20 disease condition standards covered by the questionnaire survey (S1 Table).The diseases studied include 20 conditions, consisting of 19 non-communicable diseases (hypertension, chronic digestive system diseases, heart disease, arthritis, stroke, chronic lung disease, chronic back pain, oral health disorders, diabetes, eye diseases, chronic kidney disease, thyroid disease, ear diseases, cancer, osteoporosis, anxiety, depression, epilepsy, and dementia) and one communicable disease (tuberculosis).

### Primary data collection

Data collection in Shanxi Province took place from April 14 to July 20, 2023. All study participants were required to complete a face-to-face questionnaire and undergo a physical examination. The questionnaire was based on the standard version of the Primary Healthcare Comorbidity Assessment Questionnaire (MAQ-PC) [23], and collected data on sociodemographic characteristics, lifestyle habits, and disease history. The variables included gender, age, marital status, educational level, annual family income, smoking, alcohol consumption, and physical activity. Alcohol consumption was assessed using the Alcohol Use Disorders Identification Test (AUDIT), with a total score ≥8 indicating hazardous or harmful drinking [24]. Physical activity was measured using the International Physical Activity Questionnaire (IPAQ), classifying participants into three categories: low, moderate, and high intensity [25]. The physical examination included measurements of height, weight, waist circumference, and blood pressure. A waist circumference of ≥90 cm in males and ≥85 cm in females was defined as central obesity [26]. Blood pressure was classified as ≥130/80 mm Hg [27], and BMI (weight/height²) was categorized as follows: normal (≤24 kg/m²), overweight (24–28 kg/m²), and obese (≥28 kg/m²).

### Statistical analysis

The data were independently entered by two researchers and subsequently subjected to consistency checks before forming the final database. Statistical analysis was performed using R version 4.2.1. Continuous variables were expressed as mean ± standard deviation (SD), while categorical variables were expressed as percentages. Between-group differences were analyzed using chi-square tests. The Yule’s Q method of application system cluster analysis was used to draw a dendrogram to explore differences in multimorbidity patterns between different age groups and genders. Dyad multimorbidity patterns refer to the co-existence of two disease conditions, whereas triad patterns refer to the co-existence of three disease conditions. All dyad patterns and the top ten triad multimorbidity patterns were presented, and their prevalences were calculated. All statistical analyses were considered statistically significant at P < 0.05.

## Results

### Characteristics of study participants

This study included a total of 2,208 adults, with a mean age of 57.7 ± 10.5 years. Among them, 58.1% (1,283/2,208) were 30–59 years, and 52.2% (1,152/2,208) were females. 89.7% (1,978/2,208) of participants received education up to junior high school or below, while only 10.3% (228/2,208) had an educational level of senior high school or above. A majority of participants, 90% (1,965/2,208), were married, and 49.7% (1,097/2,208) had a household annual income below 20,000 yuan. Less than two-fifths (34.4%) had a history of smoking, while more than one-tenth (13.1%) had high-risk drinking habits. The majority (74.5%) of participants engaged in high intensity physical activity. Over three-fifths (65.9%) had a BMI > 24 kg/m², and 65.3% of participants were centrally obese. 55.5% (1,226/2,208) had a systolic blood pressure (SBP) greater than 130 mm Hg, and 62.2% (1,374/2,208) had a diastolic blood pressure (DBP) greater than 80 mm Hg. Additionally, the prevalence of chronic diseases in the entire population was 69.0%, and the prevalence of local multimorbidity (simultaneous presence of two or more chronic diseases) was 37.1% (Table 1).

**Table 1:** Demographic characteristics of adult residents in rural areas of North China (N = 2208).

Characteristic	Gender		Age		Total
	Male	Female	30-59y	≥60y	
Total, n (%)	1056(47.8)	1152(52.2)	1283(58.1)	925(41.9)	2208(100)
Age (y), mean (SD)	58.1 ± 11.0	57.4 ± 10.0	50.6 ± 6.6	67.7 ± 5.7	57.7 ± 10.5
Marriage status, n (%)					
Married	917 (86.8)	1048 (91.0)	1191 (92.8)	774 (83.7)	1965 (89.0)
Widowed	72 (6.8)	92 (8.0)	31 (2.4)	133 (14.4)	164 (7.4)
Others	67 (6.3)	12 (1.0)	61 (4.7)	18 (2.0)	79 (3.6)
Educational level, n (%)					
Primary or less	392 (37.1)	622 (54.0)	491 (38.3)	523 (56.5)	1014 (45.9)
Secondary	524 (49.6)	442 (38.4)	654 (51.0)	312 (33.7)	966 (43.8)
High school and above	140 (13.3)	88 (7.6)	138 (10.8)	90 (9.7)	228 (10.3)
Annual family income (Chinese Yuan), n (%)					
<20000	528 (50.0)	569 (49.4)	464 (36.2)	633 (68.4)	1097 (49.7)
20000-50000	376 (35.6)	453 (39.3)	608 (47.4)	221 (23.9)	829 (37.6)
≥50000	152 (14.4)	130 (11.3)	211 (16.5)	71 (7.7)	282 (12.8)
Smoke status, n (%)					
Never smoked	311 (29.5)	1138 (98.8)	828 (64.5)	621 (67.1)	1449 (65.6)
Used to smoke	106 (10.0)	2 (0.2)	40 (3.1)	68 (7.4)	108 (4.9)
Now smoking	639 (60.5)	12 (1.0)	415 (32.4)	236 (25.5)	651 (29.5)
Drinking alcohol (using AUDIT), n (%)					
Low-risk drinking	769 (72.8)	1149 (99.7)	1072 (83.5)	846 (91.5)	1918 (86.9)
High-risk drinking	287 (27.2)	3 (0.3)	211 (16.5)	79 (8.5)	290 (13.1)
Physical activity (using IPAQ), n (%)					
High intensity	756 (71.6)	889 (77.2)	997 (77.7)	648 (70.1)	1645 (74.5)
Medium intensity	182 (17.2)	133 (11.5)	158 (12.3)	157 (17.0)	315 (14.3)
Low intensity	118 (11.2)	130 (11.3)	128 (10.0)	120 (13.0)	248 (11.2)
BMI (kg/m^2^), n (%)					
≤24 kg/m^2^, n (%)	408 (38.6)	411 (35.7)	434 (33.8)	385 (41.6)	819 (37.1)
24-28 kg/m^2^, n (%)	425 (40.3)	483 (41.9)	546 (42.6)	362 (39.1)	908 (41.1)
≥28 kg/m^2^, n (%)	223 (21.1)	258 (22.4)	303 (23.6)	178 (19.2)	481 (21.8)
Waist circumference, n (%)					
Normal	386 (36.5)	380 (33.0)	479 (37.3)	287 (31.0)	766 (34.7)
Central obesity	670 (63.5)	772 (67.0)	804 (62.7)	638 (69.0)	1442 (65.3)
SBP (mm Hg), n (%)					
<130 mm Hg	445 (42.1)	537 (46.6)	660 (51.4)	322 (34.8)	982 (44.5)
≥130 mm Hg	611 (57.9)	615 (53.4)	623 (48.6)	603 (65.2)	1226 (55.5)
DBP (mm Hg), n (%)					
<80 mm Hg	340 (32.2)	494 (43.0)	436 (34.0)	398 (43.0)	834 (37.8)
≥80 mm Hg	716 (67.8)	658 (57.1)	847 (66.0)	527 (57.0)	1374 (62.2)
Prevalence of Common Chronic Diseases,n (%)					
Prevalence of a single disease	712(67.42)	812 (70.49)	748 (58.30)	776 (83.89)	1524(69.02)
Prevalence of multimorbidity	325 (25.33)	493 (53.30)	376 (35.61)	442 (38.37)	818 (37.05)

Note: BMI, body mass index; SBP, Systolic blood pressure; DBP, Diastolic blood pressure; Others marriage status include divorced, separated, and unmarried.

### The prevalence of single conditions

There was no statistically significant difference in multimorbidity rates between men and women (35.6% vs. 38.4%, P > 0.05), and the multimorbidity rate was higher in those aged ≥60 years compared to those aged 30–59 years (53.3% vs. 25.3%, P < 0.001). 17.8% of participants had three or more diseases, and 7.9% had four or more diseases. There were statistically significant differences between genders (female higher than male) and age groups (≥60 years higher than 30–59 years), with the maximum number of chronic diseases reaching 10. The top five chronic diseases were Hypertension (43.5%), CDSD (12.5%), Stroke (11.4%), Arthritis (10.4%), and Diabetes (9.9%).The prevalence among individuals aged ≥60 years is higher than that of those aged 30–59 years (83.9% vs. 58.3%, *P* < 0.001). For the 30–59 age group, the prevalence is 58.3%, with hypertension (32.4%), chronic digestive system diseases (10.4%), and stroke (6.0%) being the most common diseases. For individuals aged ≥60 years, the prevalence is 83.9%, with hypertension (58.9%), stroke (18.9%), and heart disease (16.1%) being the most common diseases. There is no significant difference in prevalence between males and females (67.4% vs. 70.5%, **P* *> 0.05). The prevalence among males is 67.4%, with hypertension (42.6%), chronic digestive system diseases (10.4%), and stroke (14.2%) being the most common diseases. For females, the prevalence is 70.5%, with hypertension (44.3%), chronic digestive system diseases (14.4%), and arthritis (14.5%) being the most common diseases (Table 2).

**Table 2:** The prevalence of 20 chronic diseases in 2208 adults by gender and age.

Chronic diseases	Gender				Age				Total	
	Male(n = 1056)		Female(n = 1152)		30-59y(n = 1283)		≥60y(n = 925)		(n = 2208)	
	n	% (95% CI)	n	% (95% CI)	n	% (95% CI)	n	% (95% CI)	n	% (95% CI)
**Different number of diseases**										
Total	712	67.4(64.5,70.2)	812	70.5(67.8,73.1)	748	58.3(55.6,61.0)	776	83.9(81.2,86.1)^***^	1524	69.0(67.1,70.9)
More than two	376	35.6(32.8,38.6)	442	38.4(35.6,41.2)	325	25.3(23,27.8)	493	53.3(50.1,56.5)^***^	818	37.1(35.1,39.1)
More than three	157	14.9(12.9,17.1)	235	20.4(18.2,22.9)^***^	132	10.3(8.7,12.1)	260	28.1(25.3,31.1)^***^	392	17.8(16.2,19.4)
Four or above	62	5.9(0.46,7.4)	113	9.8(8.2,11.7)^***^	48	3.7(2.8,4.9)	127	13.7(11.7,16.1)^***^	175	7.9(6.9,9.1)
**Types of disease**										
Hypertension	450	42.6(39.7,45.6)	510	44.3(41.4,47.2)	415	32.4(29.9,35)	545	58.9(55.7,62.1)^***^	960	43.5(41.4,45.6)
CDSD	110	10.4(8.7,12.4)	166	14.4(12.5,16.6)^**^	133	10.4(8.8,12.2)	143	15.5(13.3,17.9)^***^	276	12.5(11.2,13.9)
Stroke	150	14.2(12.2,16.4)	102	8.9(7.3,10.7)^***^	77	6(4.8,7.4)	175	18.9(16.5,21.6)^***^	252	11.4(10.2,12.8)
Arthritis	62	5.9(4.6,7.4)	167	14.5(12.6,16.7)^***^	102	8(6.6,9.6)	127	13.7(11.7,16.1)^***^	229	10.4(9.2,11.7)
Diabetes	90	8.5(7.0,10.4)	129	11.2(9.5,13.2)^*^	81	6.3(5.1,7.8)	138	14.9(12.8,17.4)^***^	219	9.9(8.7,11.2)
Heart disease	118	11.2(9.4,13.2)	99	8.6(7.1,10.4)^*^	68	5.3(4.2,6.7)	149	16.1(13.9,18.6)^***^	217	9.8(8.7,11.1)
OHD	77	7.3(5.9,9.0)	104	9(7.5,10.8)	88	6.9(5.6,8.4)	93	10.1(8.3,12.2)^**^	181	8.2(7.1,9.4)
CBP	67	6.3(5.0,8.0)	88	7.6(6.2,9.3)	82	6.4(5.2,7.9)	73	7.9(6.3,9.8)	155	7.0(0.6,0.8)
Eye disease	34	3.2(2.3,4.5)	62	5.4(4.2,6.8)^*^	20	1.6(1.0,2.4)	76	8.2(6.6,10.2)^***^	96	4.4(3.6,5.3)
CLD	54	5.1(3.9,6.6)	40	3.5(2.6,4.7)	39	3(2.2,4.1)	55	6(4.6,7.7)^***^	94	4.3(3.5,5.2)
Cancer	26	2.5(1.7,3.6)	49	4.3(3.2,5.6)^*^	32	2.5(1.8,3.5)	43	4.7(3.5,6.2)^**^	75	3.4(2.7,4.2)
Ear disease	39	3.7(2.7,5.0)	28	2.4(1.7,3.5)	25	2(1.3,2.9)	42	4.5(3.4,6.1)^***^	67	3.0(2.4,3.8)
Thyroid disease	13	1.2(0.7,2.1)	49	4.3(3.2,5.6)^***^	36	2.8(2.0,3.9)	26	2.8(1.9,4.1)	62	2.8(2.2,3.6)
Osteoporosis	20	1.9(1.2,2.9)	38	3.3(2.4,4.5)	25	2(1.3,2.9)	33	3.6(2.6,5.0)^*^	58	2.6(2.0,3.4)
CKD	19	1.8(1.2,2.8)	16	1.4(0.9,2.3)	21	1.6(1.1,2.5)	14	1.5(0.9,2.5)	35	1.6(1.2,2.2)
Tuberculosis	6	0.6(0.3,1.2)	15	1.3(0.8,2.1)	12	0.9(0.5,1.6)	9	1.0(0.5,1.8)	21	1.0(0.6,1.5)
Epilepsy	7	0.7(0.3,1.4)	3	0.3(0.1,0.7)	8	0.6(0.3,1.2)	2	0.2(0.06,0.8)	10	0.5(0.2,0.8)
Anxiety	2	0.2(0.1,0.7)	7	0.6(0.3,1.3)	6	0.5(0.2,1.0)	3	0.3(0.1,0.9)	9	0.4(0.2,0.8)
Dementia	5	0.5(0.2,1.1)	3	0.3(0.1,0.7)	2	0.2(0,0.6)	6	0.7(0.3,1.4)	8	0.4(0.2,0.7)
Depression	2	0.2(0.1,0.7)	5	0.4(0.2,1.0)	6	0.5(0.2,1.0)	1	0.1(0.02,0.6)	7	0.3(0.2,0.7)

* p < 0.05, ** p < 0.01, *** p < 0.001

Note: CDSD, Chronic digestive system diseases; OHD, Oral health disorders; CLD, Chronic lung diseases; CBP, Chronic back pain; CKD, Chronic kidney diseases.

### The status of multimorbidity

Among 818 patients with multimorbidity, approximately 50% had three or more diseases, and approximately 20% had four or more diseases. There were statistically significant differences between genders (female higher than male) and age groups (≥60 years higher than 30–59 years). Among common diseases, Hypertension was the most prevalent chronic comorbid condition, accounting for 75.3%, followed by stroke (27.4%) and CDSD (26.2%).In individuals aged 30–60 years, the most common multimorbidity chronic conditions are hypertension (68.9%), chronic digestive diseases (29.2%), and arthritis (23.4%). In individuals aged ≥60 years, hypertension(79.5%), stroke(32.3%), and heart disease (24.1%)are the most prevalent multimorbidity. Among males, the most common multimorbidity are hypertension (76.9%), stroke (34.0%), and heart disease (27.1%), while among females, the most common multimorbidity are hypertension (74.0%), arthritis (31.7%), and chronic digestive diseases (29.4%), Table 3.

**Table 3:** The distribution of 20 chronic conditions among 818 cases with multimorbidity, stratified by age and gender.

Chronic diseases	Gender				Age				Total	
	Male(n = 376)		Female(n = 442)		30-59y(n = 325)		≥60y(n = 493)		(n = 818)	
	n	% (95% CI)	n	% (95% CI)	n	% (95% CI)	n	% (95% CI)	n	% (95% CI)
**Different number of diseases**										
More than three	157	41.8(36.9,46.8)	235	53.2(48.5,57.8)^***^	132	40.6(35.4,46)	260	52.7(48.3,57.1)^***^	392	47.9(44.5,51.4)
Four or above	62	16.5(13.1,20.6)	113	25.6(21.7,29.8)^**^	48	14.8(11.3,19)	127	25.8(22.1,30.0)^***^	175	21.4(18.7,24.3)
**Types of disease**										
Hypertension	289	76.9(72.3,80.8)	327	74.0(70,77.9)	224	68.9(11.3,19)	392	79.5(75.7,82.8)^***^	616	75.3(72.2,78.1)
Stroke	128	34.0(29.4,39.0)	96	21.7(18.1,25.8)^***^	65	20.0(16.0,24.7)	159	32.3(28.3,36.5)^***^	224	27.4(24.4,30.5)
CDSD	84	22.3(18.4,26.8)	130	29.4(25.4,33.8)^*^	95	29.2(24.6,34.4)	119	24.1(20.6,28.1)	214	26.2(23.3,29.3)
Arthritis	54	14.4(11.2,18.3)	140	31.7(27.5,36.2)^***^	76	23.4(19.1,28.3)	118	23.9(20.4,27.9)	194	23.7(20.9,26.8)
Heart disease	102	27.1(22.9,31.8)	91	20.6(17.1,24.6)^*^	55	16.9(13.2,21.4)	138	28(24.2,32.1)^***^	193	23.6(20.8,26.6)
Diabetes	65	17.3(13.8,21.4)	111	25.1(21.3,29.4)^**^	52	16.0(12.4,20.4)	124	25.2(21.5,29.2)^**^	176	21.5(18.8,24.5)
OHD	58	15.4(12.1,19.4)	82	18.6(15.2,22.4)	59	18.2(14.3,22.7)	81	16.4(13.4,20.0)	140	17.1(14.7,19.8)
CBP	55	14.6(11.4,18.6)	70	15.8(12.7,19.5)	62	19.1(15.2,23.7)	63	12.8(10.1,16.0)^*^	125	15.3(13.0,17.9)
Eye disease	28	7.5(5.2,10.6)	57	12.9(10.1,16.4)^*^	17	5.2(3.3,8.2)	68	13.8(11.0,17.1)^***^	85	10.4(8.5,12.7)
CLD	42	11.2(8.4,14.8)	36	8.1(5.9,11.1)	30	9.2(6.5,12.9)	48	9.7(7.4,12.7)	78	9.5(7.7,11.8)
Ear disease	32	8.5(6.1,11.8)	23	5.2(3.5,7.7)	16	4.9(3.1,7.8)	39	7.9(5.8,10.6)	55	6.7(5.2,8.7)
Osteoporosis	19	5.1(3.3,7.8)	36	8.1(5.9,11.1)	24	7.4(5.0,10.8)	31	6.3(4.5,8.8)	55	6.7(5.2,8.7)
Cancer	16	4.3(2.6,6.8)	36	8.1(5.9,11.1)^*^	16	4.9(3.1,7.8)	36	7.3(5.3,9.9)	52	6.4(4.9,8.2)
Thyroid disease	9	2.4(1.3,4.5)	36	8.1(5.9,11.1)^***^	22	6.8(4.5,10)	23	4.7(3.1,6.9)	45	5.5(4.1,7.3)
CKD	16	4.3(2.6,6.8)	12	2.7(1.6,4.7)	16	4.9(3.1,7.8)	12	2.4(1.4,4.2)	28	3.4(2.4,4.9)
Tuberculosis	6	1.6(0.07,3.4)	11	2.5(1.4,4.4)	10	3.1(1.7,5.6)	7	1.4(0.7,2.9)	17	2.1(1.3,3.3)
Anxiety	2	0.5(0.02,1.9)	7	1.6(0.8,3.2)	6	1.9(0.9,4.0)	3	0.6(0.2,1.8)	9	1.1(0.6,2.1)
Dementia	4	1.1(0.4,2.7)	3	0.7(0.02,2.0)	1	0.3(0.1,1.7)	6	1.2(0.6,2.6)	7	0.9(0.4,1.8)
Depression	2	0.5(0.02,1.9)	5	1.1(0.5,2.6)	6	1.9(0.9,4.0)	1	0.2(0.03,1.1)^*^	7	0.9(0.4,1.8)
Epilepsy	4	1.1(0.4,2.7)	1	0.2(0.04,1.3)	3	0.9(0.3,2.7)	2	0.4(0.1,1.5)	5	0.6(0.3,1.4)

* p < 0.05, ** p < 0.01, *** p < 0.001

Note: CDSD, Chronic digestive system diseases; OHD, Oral health disorders; CLD, Chronic lung diseases; CBP, Chronic back pain; CKD, Chronic kidney diseases.

### Multimorbidity patterns

In this study, the top three dyad multimorbidity patterns are hypertension and stroke (23.3%), hypertension and heart disease (18.5%), and hypertension and chronic digestive system diseases (17.0%). The most common triad multimorbidity patterns is hypertension and stroke and heart disease (5.5%). Among different populations, the top three dyad multimorbidity patterns are as follows, in the 30–59 age group, they are hypertension and stroke (16.9%), hypertension and chronic digestive system diseases (16.3%), and hypertension and arthritis (14.5%). in the ≥ 60 age group, they are hypertension and stroke (27.6%), hypertension and heart disease (23.1%), and hypertension and diabetes (19.9%). For males, the most common dyad multimorbidity patterns are hypertension and stroke (29.3%), hypertension and heart disease (21.0%), and hypertension and chronic digestive system diseases (13.3%). for females, they are hypertension and arthritis (21.9%), hypertension and chronic digestive system diseases (diabetes) (20.1%), and hypertension and stroke(18.3%), Table 4.

**Table 4:** Top ten frequent unique combination clusters with multimorbidity, stratified by age and gender.

Factor	Dyads of morbidity			Triads of morbidity		
	Combination	n	% (95% CI)	Combination	n	% (95% CI)
**Total(n = 818)**						
	Stroke & Hypertension	191	23.3(20.1,25.9)	Stroke & Heart disease & Hypertension	45	5.5(4.4,7.6)
	Heart disease & Hypertension	151	18.5(15.4,20.6)	Diabetes & Stroke & Hypertension	35	4.3(2.7,5.3)
	CDSD & Hypertension	139	17(14.4,19.6)	Stroke & Arthritis & Hypertension	33	4.0(2.7,5.3)
	Diabetes & Hypertension	138	16.9(14.4,19.6)	Heart disease & CDSD & Hypertension	33	4.0(2.7,5.3)
	Arthritis & Hypertension	132	16.1(13.5,18.5)	Arthritis & CDSD & Hypertension	32	3.9(2.7,5.3)
	OHD & Hypertension	85	10.4(7.9,12.1)	Diabetes & Heart disease & Hypertension	31	3.8(2.7,5.3)
	CBP & Hypertension	70	8.6(7.0,11.0)	Stroke & CDSD & Hypertension	31	3.8(2.7,5.3)
	Eye disease & Hypertension	60	7.3(5.3,8.7)	CBP & CDSD & Hypertension	30	3.7(2.7,5.3)
	Arthritis & CDSD	56	6.8(5.3,8.7)	CBP & Arthritis & Hypertension	29	3.5(2.7,5.3)
	CLD & Hypertension	53	6.5(4.4,7.6)	Diabetes & Arthritis & Hypertension	28	3.4(1.8,4.2)
**30-60y(n = 325)**						
	Stroke & Hypertension	55	16.9(12.9,21.1)	Arthritis & CDSD & Hypertension	12	3.7(1.9,6.1)
	CDSD & Hypertension	53	16.3(12.0,20.0)	Stroke & CDSD & Hypertension	12	3.7(1.9,6.1)
	Arthritis & Hypertension	47	14.5(10.2,17.8)	CBP & CDSD & Hypertension	11	3.4(1.1,4.9)
	Diabetes & Hypertension	40	12.3(8.5,15.5)	CBP & Arthritis & Hypertension	9	2.8(1.1,4.9)
	Heart disease & Hypertension	37	11.4(7.6,14.4)	OHD & Stroke & Hypertension	9	2.8(1.1,4.9)
	OHD & Hypertension	31	9.5(6.7,13.3)	Heart disease & CDSD & Hypertension	8	2.5(0.5,3.5)
	CBP & Hypertension	28	8.6(5.9,12.1)	Diabetes & Arthritis & Hypertension	7	2.2(0.5,3.5)
	Arthritis & CDSD	25	7.7(5.1,10.9)	CBP & Arthritis & CDSD	7	2.2(0.5,3.5)
	CBP & CDSD	24	7.4(4.2,9.8)	Thyroid disease & CDSD & Hypertension	7	2.2(0.5,3.5)
	CBP & Arthritis	17	5.2(2.6,7.4)	Stroke & Arthritis & Hypertension	6	1.8(0.5,3.5)
**≥60(n = 493)**						
	Stroke & Hypertension	136	27.6(24.0,32.0)	Stroke & Heart disease & Hypertension	39	7.9(5.6,10.4)
	Heart disease & Hypertension	114	23.1(19.3,26.7)	Diabetes & Stroke & Hypertension	31	6.3(3.9,8.1)
	Diabetes & Hypertension	98	19.9(16.5,23.5)	Stroke & Arthritis & Hypertension	27	5.5(3.1,6.9)
	CDSD & Hypertension	86	17.4(13.7,20.3)	Diabetes & Heart disease & Hypertension	26	5.3(3.1,6.9)
	Arthritis & Hypertension	85	17.2(13.7,20.3)	Heart disease & CDSD & Hypertension	25	5.1(3.1,6.9)
	OHD & Hypertension	54	11.0(8.2,13.8)	Diabetes & Arthritis & Hypertension	21	4.3(2.3,5.7)
	Eye disease & Hypertension	50	10.1(7.4,12.6)	Arthritis & CDSD & Hypertension	20	4.1(2.3,5.7)
	Stroke & Heart disease	45	9.1(6.5,11.5)	CBP & Arthritis & Hypertension	20	4.1(2.3,5.7)
	CBP & Hypertension	42	8.5(6.5,11.5)	Stroke & CDSD & Hypertension	19	3.9(2.3,5.7)
	Diabetes & Stroke	37	7.5(5.6,10.4)	CBP & CDSD & Hypertension	19	3.9(2.3,5.7)
**Male(n = 376)**						
	Stroke & Hypertension	110	29.3(24.4,33.6)	Stroke & Heart disease & Hypertension	24	6.4(3.6,8.4)
	Heart disease & Hypertension	79	21.0(16.9,25.1)	Diabetes & Stroke & Hypertension	18	4.8(2.8,7.2)
	CDSD & Hypertension	50	13.3(9.6,16.4)	Diabetes & Heart disease & Hypertension	15	4.0(2.0,6.0)
	Diabetes & Hypertension	49	13.0(9.6,16.4)	Heart disease & CDSD & Hypertension	12	3.2(1.3,4.7)
	Arthritis & Hypertension	35	9.3(6.1,11.9)	Stroke & Arthritis & Hypertension	11	2.9(1.3,4.7)
	OHD & Hypertension	33	8.8(6.1,11.9)	CBP & Arthritis & Hypertension	11	2.9(1.3,4.7)
	CBP & Hypertension	31	8.2(5.3,10.7)	CBP & CDSD & Hypertension	11	2.9(1.3,4.7)
	Stroke & Heart disease	29	7.7(5.3,10.7)	CBP & Stroke & Hypertension	10	2.7(1.3,4.7)
	CLD & Hypertension	28	7.4(4.4,9.6)	Stroke & CDSD & Hypertension	9	2.4(0.6,3.4)
	Diabetes & Stroke	22	5.9(3.6,8.4)	CLD & Heart disease & Hypertension	9	2.4(0.6,3.4)
**Female(n = 442)**						
	Arthritis & Hypertension	97	21.9(18.1,25.9)	Arthritis & CDSD & Hypertension	26	5.9(3.8,8.2)
	CDSD & Hypertension	89	20.1(16.3,23.7)	Diabetes & Arthritis & Hypertension	24	5.4(3.0,7.0)
	Diabetes & Hypertension	89	20.1(16.3,23.7)	Stroke & Arthritis & Hypertension	22	5.0(3.0,7.0)
	Stroke & Hypertension	81	18.3(14.4,21.6)	Stroke & CDSD & Hypertension	22	5.0(3.0,7.0)
	Heart disease & Hypertension	72	16.3(12.6,19.4)	Stroke & Heart disease & Hypertension	21	4.8(3.0,7.0)
	OHD & Hypertension	52	11.8(9.0,15.0)	Heart disease & CDSD & Hypertension	21	4.8(3.0,7.0)
	Eye disease & Hypertension	41	9.3(6.3,11.7)	CBP & CDSD & Hypertension	19	4.3(2.2,5.8)
	Arthritis & CDSD	41	9.3(6.3,11.7)	Arthritis & Heart disease & Hypertension	19	4.3(2.2,5.8)
	CBP & Hypertension	39	8.8(6.3,11.7)	CBP & Arthritis & Hypertension	18	4.1(2.2,5.8)
	Diabetes & Arthritis	33	7.5(4.6,9.4)	Diabetes & Stroke & Hypertension	17	3.8(2.2,5.8)

Note: CDSD, Chronic digestive system diseases; OHD, Oral health disorders; CLD, Chronic lung diseases; CBP, Chronic back pain; CKD, Chronic kidney diseases.

A systematic cluster analysis was conducted using the 20 chronic diseases included in the study as cluster indicators, with Yule’s Q method employed to generate a cluster dendrogram. In the general population, the first cluster included Depression, Anxiety, Osteoporosis, and Thyroid disease; the second cluster comprised CLD, Tuberculosis, OHD, and Cancer; the third cluster included Arthritis, CBP, CDSD, Hypertension, Stroke, Heart disease, Ear disease, Dementia, Eye disease, CKD, and Diabetes; and the fourth cluster was composed of Epilepsy, S1 Fig.

## Discussion

In this post-hoc exploratory analysis, we found that nearly 70% (69.0%) of individuals aged 30 and above in rural areas of Shanxi Province, China, had at least one disease, and over one-third (37.1%) had two or more diseases. We also found that, regardless of age or gender, the primary multimorbidity pattern was associated with hypertension. However, the multimorbidity patterns varied across different gender and age groups.

This study found that the prevalence of chronic diseases in rural areas of Shanxi Province is higher than that of the population aged 35 and above in rural areas of Southwest China in 2021 (50.0%) [28].In addition, the prevalence of multimorbidity in rural Shanxi Province was also higher than the 25.4% in a meta-analysis of Chinese adults over the past decade [29], but lower than the 44.4% global prevalence in a global meta-analysis of people aged 30 years and older [5].Particularly, the prevalence of chronic diseases among individuals aged 30–60 is as high as 58.3%, and this group is expected to become the primary source of future multimorbidity. The prevalence of chronic diseases is increasing significantly, particularly the high prevalence (83.89%) observed among individuals aged 60 and above in this study. This trend may be attributed to multiple factors: improved economic conditions have led to increasingly abundant and sophisticated diets, but the lack of scientific nutritional guidance in rural areas has caused a greater energy intake–expenditure imbalance [30,31]. Meanwhile, the advancement of agricultural mechanization and pervasive use of electronic devices in daily life have reduced physical activity levels [7]. Additionally, improved healthcare access has prolonged life expectancy, further contributing to this trend [32,33]. It is important to note that the inclusion of diverse disease types and conventional data collection methods in this study may have led to an overestimation of the actual disease prevalence. The prevalence of multimorbidity increases with age among individuals aged 60 and above [29], which is consistent with previous research. For females, hormonal changes may contribute to an increased risk of chronic diseases [34].

Multiple diseases may originate from similar genetic backgrounds or environmental triggers, or share common pathogenic mechanisms, thereby exhibiting a tendency to co-occur and ultimately forming specific multimorbidity patterns. The multimorbidity patterns found in this study are similar to those observed in Asian systematic reviews [35], indicating that cardiovascular and metabolic diseases (such as hypertension, diabetes, stroke, dyslipidemia, and heart disease) are common patterns of multimorbidity. Hypertension is the primary multimorbidity in rural areas of China, consistent with previous studies [36], and is likely closely related to high-salt diets and a lack of fresh fruits and vegetables [37]. It is also considered a risk factor for other more severe diseases, such as stroke, diabetes, and chronic heart disease [38], highlighting the importance of managing hypertension in chronic disease management [39].

A systematic review of 51 studies on comorbidity patterns revealed that the clustering of mental health disorders and cardiovascular metabolic diseases is highly prevalent, with osteoporosis, back pain, musculoskeletal disorders, and soft tissue disorders being common clustered conditions [40]. This aligns with the common disease clusters observed in the general population of this study. Additionally, the frequent clustering of thyroid diseases and osteoporosis may be related to the negative correlation between thyroid hormone levels and bone mass, bone density, and muscle mass [41]. This study found that the most common multimorbidity patterns in individuals aged 30–59 were hypertension combined with chronic digestive system diseases, stroke, and arthritis. For those aged 60 and above, the predominant pattern was hypertension with stroke, heart disease, and diabetes. This differs from the patterns reported by Geng et al [42] for individuals under 60, where the leading conditions were dyslipidemia, obesity, hypertension, cervical and lumbar spine diseases, and digestive system diseases. The higher prevalence of chronic digestive diseases in the 30–59 age group in this study may be closely related to factors such as irregular dietary habits, lack of physical activity, and high levels of stress, these factors are risk factors for hypertension, diabetes, heart disease, and stroke [43]. Particularly, the study area is known for a high prevalence of esophageal cancer and other digestive system cancers [44], which may involve certain genetic factors. In addition, as industrialization, urbanization, and urban population growth have exacerbated air pollution, there is a correlation between exposure to air pollution and a higher incidence of digestive system diseases [45]. The frequent coexistence of stroke in this group aligns with the trend of younger onset, potentially driven by the high prevalence of risk factors such as hypertension, hyperlipidemia, obesity, and smoking among the younger population [46]. The occurrence of arthritis may be linked to the cold climate in northern rural areas, prolonged exposure to low sunlight, and a limited diet, which can result in insufficient intake of calcium and other trace elements [47]. In individuals aged 60 and above, the accumulation of diseases related to the circulatory and metabolic systems, which are typical of aging, is more prevalent [48,49].This study found that in the male population, the most common multimorbidity pattern is hypertension combined with stroke, heart disease, and chronic digestive system diseases. In the female population, the pattern is hypertension combined with arthritis, diabetes, and chronic digestive system diseases. Compared to the study by Wang et al [50], which shows that the multimorbidity patterns in males are mainly heart disease, glycoprotein metabolism disorders, hypertension, lipoprotein metabolism disorders, and stroke, while females primarily show combinations of heart disease, lipoprotein metabolism disorders, spinal diseases, stroke, and hypertension, there are some differences. The potential reasons for these differences are as follows. First, unhealthy lifestyle habits are important behavioral risk factors for cardiovascular diseases in males in Shanxi Province [51], such as poor diet, smoking, and drinking. Second, the risk of chronic diseases such as arthritis and diabetes is higher in females than in males [52,53], and the effects of changes in estrogen levels and psychosocial risk factors are more pronounced in females [54]. Finally, studies have shown that long-term use of solid cooking fuels carries a higher risk of chronic digestive disorders [55], which explains why digestive disorders are even higher among females than males.

## Strengths and limitations

The main strengths of this study are as follows. First, participants were stratified by age and gender to maximize the representativeness of the study sample within the research area. Second, routine data collection methods were used to complement self-reported data, thereby reducing potential recall bias and ensuring both the completeness of the data and the reliability of the results. Finally, the prevalence of multimorbidity varies across countries and regions, influenced by factors such as geographical environment, lifestyle, and healthcare resources [56]. For example, in the United States, the characteristics of multimorbidity primarily involve metabolic diseases [57], while in France, in addition to metabolic diseases, mental health issues are also prevalent [58]. In contrast, this study covers a wide range of diseases, including hypertension, diabetes, digestive disorders, respiratory conditions, musculoskeletal diseases, and mental health issues, providing a comprehensive assessment of the composition of multimorbidity. However, there are several limitations in this study. Firstly, the migration of individuals aged 30–59 from rural areas, despite the stratification by age and gender, resulted in an average age for this group that is close to its upper limit, potentially limiting the accuracy in reflecting the multimorbidity and composition patterns of younger populations. Additionally, the low health literacy among rural residents in China may result in participants being unaware of their health status, potentially leading to an underestimation of the prevalence of the target diseases. Finally, although the study focused on populations from two counties in Shanxi Province and employed cluster random sampling, the generalizability of the findings may still be limited.

## Conclusion

Multimorbidity has emerged as a prevailing health pattern among adults in rural China, with hypertension being the most preponderant condition in complex constellations of multiple chronic diseases. The focus on specific diseases varies across different population groups, suggesting the need for stratified, targeted, and standardized management strategies. For instance, among individuals aged 30–59, priority should be given to cardiovascular diseases and chronic digestive system disorders, while for those aged 60 and above, the focus should shift to preventing diabetes in conjunction with cardiovascular conditions. In men, the emphasis should be placed on cardiovascular diseases and chronic digestive disorders, whereas in females, the focus should be on hypertension and arthritis.

## Supporting information

S1 Fig Cluster analysis of multimorbidity patterns in 20 chronic disease (Total). (TIF)

S1 Table ICD-10 codings of the 20 disease conditions collected in the questionnaire. (DOCX)
