# Supplementary figures and images for: Prevalence and patterns of multimorbidity among adults in rural Shanxi Province, China: A post-hoc exploratory subgroup analysis of a cross-sectional study
Source: PLoS One. 2025 Sep 10;20(9):e0330935. doi: 10.1371/journal.pone.0330935 (PMC12422502; doi:10.1371/journal.pone.0330935)

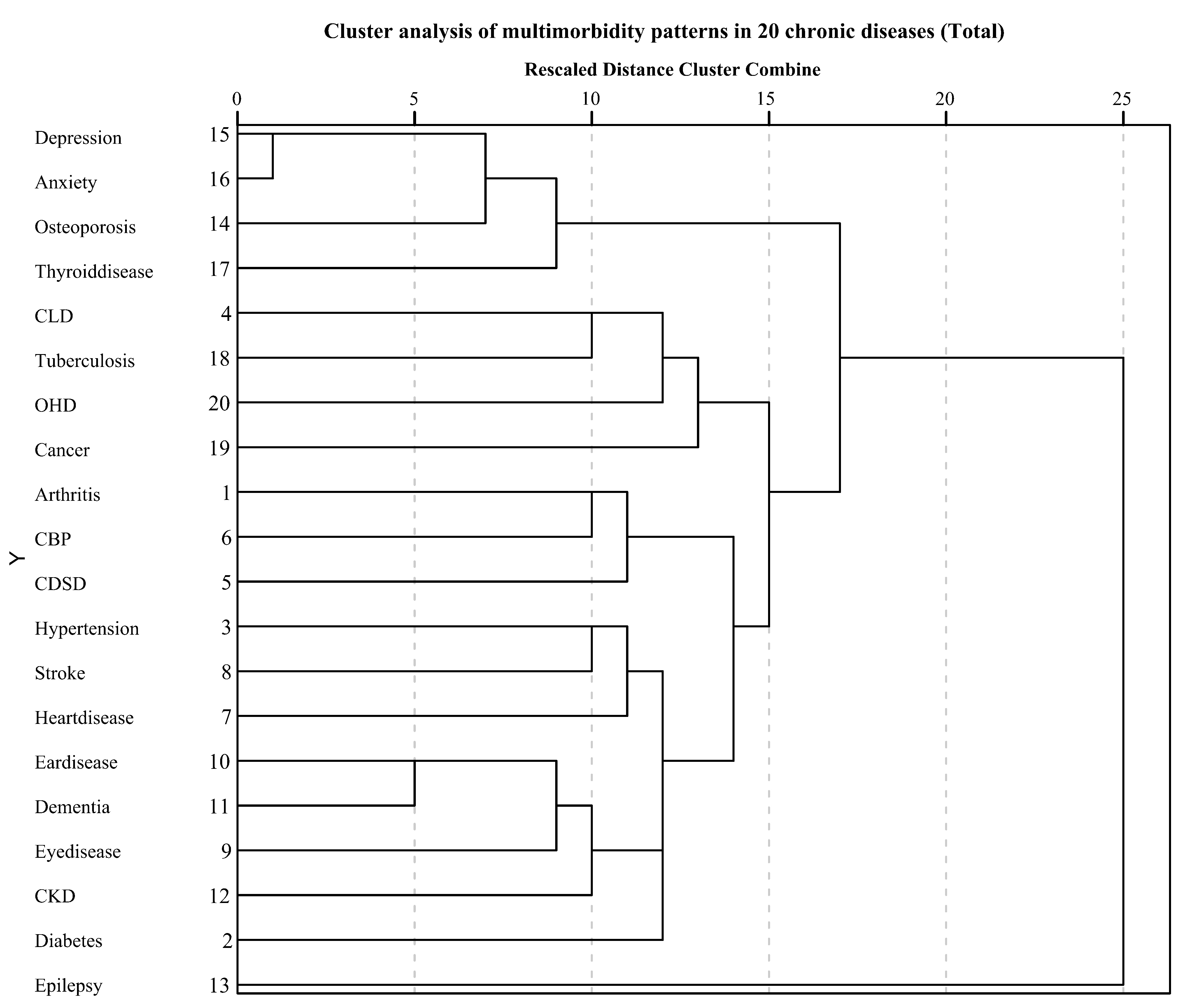

Supplement: S1 Fig — (TIF) [file pone.0330935.s001.tif]
