# Supplementary material for: Prevalence and patterns of multimorbidity among adults in rural Shanxi Province, China: A post-hoc exploratory subgroup analysis of a cross-sectional study
Source: PLoS One. 2025 Sep 10;20(9):e0330935. doi: 10.1371/journal.pone.0330935 (PMC12422502; doi:10.1371/journal.pone.0330935)
Supplement: S1 Table — (DOCX) [file pone.0330935.s002.docx]

| S1 Table. ICD-10 codings of the 20 disease conditions collected in the questionnaire. | | | |
| --- | --- | --- | --- |
| NO. | Twenty Diseases or health conditions included in the study | Disease category (ICD-10 chapter) | ICD-10 code |
| 1 | Hypertension | Hypertensive diseases | I10-I15 |
| 2 | Chronic digestive system diseases | Diseases of oesophagus, stomach and duodenum | K25-K29, K31 |
| 3 | Heart disease | Chronic rheumatic heart diseases | I05-I09 |
|  |  | Ischemic heart diseases | I20-I25 |
|  |  | Hypertensive Heart Disease | I11 |
|  |  | Pulmonary Heart Disease | I26-I28 |
| 4 | Arthritis | Rheumatoid Arthritis | M05, M06 |
|  |  | Osteoarthritis | M15-M19 |
| 5 | Stroke | Cerebrovascular diseases | I60-I69 |
| 6 | Chronic lung disease | Chronic obstructive bronchitis | J43-J44, J47 |
|  |  | Asthma | J45-J46 |
| 7 | Chronic back pain | Dorsalgia | M54 |
| 8 | Oral health disorders | Diseases of pulp and periapical tissues | K04 |
|  |  | Gingival and Periodontal Disorders | K05 |
| 9 | Diabetes | Type 2 diabetes mellitus | E11 |
| 10 | Eye disease | Disorders of lens | H25-H28 |
|  |  | Disorders of choroid and retina | H30-H36 |
|  |  | Disorders of sclera, cornea, iris and ciliary body | H15-H22 |
|  |  | Glaucoma | H40-H42 |
| 11 | Chronic kidney disease | Chronic Kidney Disease | N18 |
| 12 | Thyroid disease | Hypothyroidism | E03 |
|  |  | Hyperthyroidism, Thyrotoxicosis | E05 |
| 13 | Ear disease | Diseases of middle ear and mastoid | H65-H75 |
|  |  | Diseases of inner ear | H80-H83 |
|  |  | Other disorders of ear | H90-H95 |
| 14 | Cancer | Malignant neoplasm of oesophagus | C15 |
|  |  | Malignant neoplasm of stomach | C16 |
|  |  | Malignant neoplasm of colon | C18-C20 |
|  |  | Malignant neoplasm of liver and intrahepatic bile ducts | C22 |
|  |  | Malignant neoplasm of pancreas | C25 |
|  |  | Malignant neoplasm of bronchus and lung | C34 |
|  |  | Malignant neoplasm of breast | C50 |
|  |  | Malignant neoplasm of cervix uteri | C53 |
|  |  | Malignant neoplasm of prostate | C61 |
|  |  | Malignant neoplasm of kidney, except renal pelvis | C64 |
| 15 | Osteoporosis | Disorders of bone density and structure | M80-M82 |
| 16 | Anxiety | Phobic anxiety disorders | F40 |
|  |  | Other anxiety disorders | F41 |
| 17 | Depression | Depressive episode | F32 |
|  |  | Recurrent depressive disorder | F33 |
| 18 | Epilepsy | Epilepsy | G40 |
| 19 | Dementia | Alzheimer's disease | G30 |
|  |  | Dementia in Alzheimer's disease | F00 |
|  |  | Dementia in Other Diseases | F02 |
| 20 | Tuberculosis | Respiratory tuberculosis, bacteriologically and histologically confirmed | A15 |
